# The Co-Morbidity between Bipolar and Panic Disorder in Fibromyalgia Syndrome

**DOI:** 10.3390/jcm9113619

**Published:** 2020-11-10

**Authors:** Alessandra Alciati, Fabiola Atzeni, Daniela Caldirola, Giampaolo Perna, Piercarlo Sarzi-Puttini

**Affiliations:** 1 Department of Clinical Neurosciences, Hermanas Hospitalarias, Villa San Benedetto Menni Hospital, Albese con Cassano, via Roma 16, 22032 Como, Italy; caldiroladaniela@gmail.com (D.C.); pernagp@gmail.com (G.P.); 2 Department of Biomedical Sciences, Humanitas University, Via Rita Levi Montalcini 4, 20090 Pieve, Emanuele-Milan, Italy; 3 Rheumatology Unit, Department of Internal Medicine, University of Messina, Via Consolare Valeria 1, 98100 Messina, Italy; atzenifabiola@hotmail.com; 4 Department of Psychiatry and Neuropsychology, Faculty of Health, Medicine and Life Sciences, University of Maastricht, 6200 Maastricht, The Netherlands; 5 Department of Psychiatry and Behavioral Sciences, Leonard Miller School of Medicine, University of Miami, Miami, FL 33136-1015, USA; 6 Rheumatology Unit, Internal Medicine Department, ASST Fatebenefratelli-Sacco, Via GB Grassi 74, 20157 Milan, Italy; piercarlo.sarziputtini@gmail.com

**Keywords:** bipolar disorders, panic disorder, fibromyalgia, co-morbidity

## Abstract

About half of the patients with fibromyalgia (FM) had a lifetime major depression episode and one third had a panic disorder (PD). Because the co-morbidity between bipolar disorder (BD) and PD marks a specific subtype of BD we aimed to investigate if co-morbid BD/PD (comBD/PD) occurs more frequently than the single disorder in FM patients and evaluate the clinical significance and timing of this co-morbidity. Further, we explored the role of co-morbid subthreshold BD and PD. In 118 patients with FM, lifetime threshold and sub-threshold mood disorders and PD were diagnosed with Diagnostic and Statistical Manual of Mental Disorders-IV-Text Revision (DSM-IV-TR) Clinical Interview. Demographic and clinical variables were compared in co-morbid BD/PD (comBD/PD) and not co-morbid BD/PD (nocomBD/PD) subgroups. The co-morbidity BD/PD was seen in 46.6% of FM patients and in 68.6% when patients with minor bipolar (MinBD) and sub-threshold panic were included. These rates are higher than those of the general population and BD outpatients. There were no statistically significant differences between threshold and sub-threshold comBD/PD and nocom-BD/PD subgroups in demographic and clinical parameters. In the majority of patients (78.2%), the onset of comBD/PD preceded or was contemporary with FM. These findings support the hypothesis that comBD/PD is related to the development of FM in a subgroup of patients.

## 1. Introduction

Fibromyalgia (FM) is a syndrome of unknown etiology characterized by widespread muscle-skeletal pain lasting for more than three months and a range of other symptoms that generally include, but are not limited to persistent fatigue, sleep disturbance, and cognitive dysfunction. Co-morbid depression and anxiety symptoms and disorders are common. About half of patients with FM have a lifetime major depressive episode [1,2,3] and up to 90% reported depressive symptoms [4]. Several studies [5,6] demonstrated that, in patients with FM, major depression frequently occurs in the context of bipolar spectrum disorder (BSD), being accompanied by hypomanic syndrome or symptoms, which corresponds to the Diagnostic and Statistical Manual of Mental Disorders, Fifth Edition (DSM-5) diagnostic categories of bipolar II disorder (BD-II) and other specified bipolar or related disorder, respectively. In particular, evidence suggests an increased prevalence of bipolar II compared to bipolar I (BD-I) disorder in FM patients [5,7] and high frequency of manic symptoms (59%) which approximately double that found in healthy controls from the general population [8].

Relative to depressive symptoms and disorders, the assessment of anxiety in patients with FM has received less attention. On average, anxiety symptoms have been reported in forty-five percent of FM patients [9,10] while anxiety disorders have been diagnosed more than in one third [1,2,11]. The lifetime prevalence of panic disorder (PD), one of the most disabling anxiety disorders, ranged from 17% to 72% in patients with FM [8,9,10], while it has been detected in 3–4% of the general population [12].

In addition to full-blown mood and anxiety disorders, studies described a high prevalence, in FM patients, of subthreshold psychopathology and its association with greater physical impairment, poorer functioning, and lower health-related quality of life [4,13,14]. Subthreshold mood and anxiety disorders are also highly prevalent in patients with functional gastrointestinal disorders, such as functional dyspepsia and irritable bowel syndrome [15], which frequently co-occur with FM and likely share the same pathogenetic mechanisms. These findings suggest a role of subthreshold psychopathology in FM and functional disorders. Of note, it is thought that FM symptoms and depressive/anxiety symptoms affect each other and have a reciprocal mutual enhancing influence. In particular pain and disabilities increase depression and anxiety, which in turn may aggravate the primary symptoms of FM (pain, fatigue, and insomnia) and reduce health-related quality of life.

The literature has provided compelling evidence that anxiety disorders (ADs) are the most prevalent psychiatric co-morbidity among patients with bipolar disorder (BD) [16]. The co-morbidity between BD and ADs have been widely associated with negative outcomes, in particular high co-morbidity with psychiatric and physical illnesses, greater severity and frequency of mood episodes, suicidal behavior [17], and less favorable response to pharmacological or psychological treatments [18].

A random-effect meta-analysis [19] indicated that PD is the most common co-morbid anxiety disorder in BD with a prevalence of 16.8%. Both community-based [20] and clinical studies [21,22] have supported the link between BD and PD. Similarly, among outpatients with PD, the rates of BD and BSD (sub-threshold BD/soft BD) are quite higher than the population prevalence [23].

The increased prevalence of PD among BD subjects seems to involve individuals with bipolar II disorder (BIP-II) [24]. One of the most striking findings of the Bridge Study (which recruited 5635 patients from 18 countries presenting for treatment with major depressive episodes), is that patterns of concurrent co-morbidities differed significantly between BD-I and BD-II patients. In particular, BD-II, especially if diagnosed with a revised set of diagnostic criteria under the diagnostic threshold of DSM-IV and International Classification of Diseases 10th Revision (ICD-10), have shown a clearly higher co-morbidity with PD (in particular in females) than BD-I and major depression disorder (MDD) [25]. 

The high rates of co-occurrence between BD and PD among families [26,27] have suggested that BD with co-morbid PD can represent a genetic subtype of BD. A linkage of BD to chromosome 18q, in particular in patients with BD-II [28], has been reported [29,30].

The evidence described above led us to hypothesize that the epidemiological connection between BP and PD can express also in the context of FM, in which these two disorders are individually highly prevalent. Because the available literature focused on depressive and anxiety co-morbidity of FM only considering the single disorder at a time, the primary aim of this observational, cross-sectional study was to assess the association between BD and PD, its demographic and clinical correlates as well as the temporal relationship among BD, PD, and FM, in a population of patients with FM attending to a tertiary care center. Subthreshold PD is much less studied than subthreshold BD/BSD in clinical samples and official nosographic criteria for its diagnosis are still unavailable. However, population-based studies have demonstrated that a sizeable proportion of individuals with subthreshold PD had an unfavorable prognosis [31] and elevated comorbidity with psychiatric pathology, in particular functional /somatoform disorders [32], among which many patients with FM may be included. For these reasons, a secondary aim of our study is to perform an exploratory analysis of the association between the co-morbid threshold and subthreshold mood disorders and PD in patients with FM.

## 2. Methods

### 2.1. Subjects

The study involved 118 outpatients consecutively referring to the Rheumatology Department of L. Sacco University Hospital in Milan, Italy, between May 2010 and May 2011. Part of this sample was examined in previous studies that had a different aim [5].

Inclusion criteria were (a) age: 18–70 years; (b) to meet the 1990 American College of Rheumatology criteria for fibromyalgia requiring widespread pain, for at least 3 months, above and below the waist on both sides of the body and on axial skeleton, as well as pain present in 11 or more of 18 “tender points” detected by a pressure of 4 kg/cm^2^ applied for a few seconds [33]. In addition, axial skeletal pain (cervical spine or anterior chest or thoracic spine or low back) must be present.

Exclusion criteria were (a) inflammatory causes of pain, (b) severe and uncontrolled medical illnesses, (c) lifetime neurological disorders, (d) alcohol/cocaine abuse or dependence, (e) any clinical condition that may affect the reliability of the assessment, and (f) pregnancy.

All subjects provided written informed consent after receiving a complete description of the study. The research was approved by the ethics committee at the L. Sacco University Hospital, Milan, Italy on 8 July 2010 (No. 293/2010/26/AP).

### 2.2. Procedure

During a rheumatological visit, the subjects were asked whether they were willing to undergo a psychiatric assessment in the framework of a research study. Clinical and socio-demographic data were collected using interviewer-administered questionnaires and recorded through a structured interview format; when possible, the data were validated utilizing medical records and interviews of family members. The validation of interview data with collateral sources of information was done in about 70% of cases. 

### 2.3. Psychiatric Diagnoses

#### 2.3.1. Mood Disorders

Lifetime psychiatric diagnoses were made with the Structured Clinical Interview for DSM-IV-TR (SCID) Axis I Disorders, Mood Disorders modules [34] in a cross-sectional, single assessment. As the BSD is highly prevalent in individuals with FM [5,6] subjects with a sub-threshold expression of bipolarity were also recruited. In order to improve the detection of subthreshold pathology [35], modified DSM-IV-TR criteria (Zurich criteria) were used [36]. Zurich criteria mainly broaden the DSM-IV-TR criteria for hypomania, introducing the following adjustments: (1) the increased goal-directed activity was considered a stem criterion (this change was later included in the DSM-5 diagnostic criteria for hypomania); (2) the DSM-IV-TR 4-day minimum duration was not adhered to and patients with hypomania lasting for at least one day were included, based on studies showing close similarities in diagnostic validators between BD-II with short periods of hypomania (1–3 days) and DSM-IV-TR bipolar disorder [36].

We applied the following diagnoses of mood disorders:

#### 2.3.2. Definition of the Bipolar Spectrum Disorders

Major bipolar spectrum

(1) Bipolar I disorder (BD-I): major depressive episode associated with a manic syndrome.

(2) Bipolar II disorder (BD-II): major depressive episode associated with (a) a hypomanic syndrome or (b) a subsyndromal hypomanic episode (both defined below). For the analyses, these subjects were grouped.

Minor bipolar spectrum

(3) Minor bipolar disorder (MinBD): dysthymia, minor depression or recurrent brief depression associated with (a) a hypomanic syndrome, or (b) hypomanic symptoms only (subsyndromal hypomanic episode). 

The hypomanic syndrome was defined according to the Zurich criteria (36): (1) euphoria, irritability, or overactivity; (2) patients have themselves experienced problems or received comments from others that something must be wrong with them (consequences); and (3) patients presented at least three out of seven signs and symptoms of DSM-IV hypomania.

The subsyndromal hypomanic episode was defined as an episode of at least two hypomanic symptoms, which did not meet DSM-IV criteria for hypomania (number of symptoms below DSM-IV cut-off, or mood changes not present, or duration of ≥1 day) and did not have consequences [36,37].

Recurrent brief depression was diagnosed according to ICD-10. Minor depression was diagnosed according to the Zurich definition [36], which required three to four of nine DSM-III-R criterial symptoms, with a minimum duration of 2 weeks. 

Measure of lifetime hypomanic symptoms

Hypomania Symptom Checklist

The Hypomania Symptom Checklist (HCL-32) is a self-administered inventory consisting of 32 yes/no items used to identify possible symptoms of hypomania in clinical and non-clinical samples [38].

The analyses of the HCL responses in this paper are restricted to the checklist of hypomanic items. The total HCL-32 score was obtained by summing all items rated “yes”. The cut-off score of 14 has been accepted as optimal for discriminating BD from MDD in previous studies [39].

#### 2.3.3. Definition of Unipolar Spectrum Diagnoses

Major unipolar spectrum

Major depression disorder (MDD), single or recurrent episodes

Minor unipolar spectrum 

Dysthymia, minor depression or recurrent brief depression 

The “no” mood disorder (none) category implies no bipolar or unipolar disorder only.

#### 2.3.4. Panic Disorders

Definition of Panic Disorder

PD was diagnosed according to DSM-IV criteria as determined by the Structured Clinical Interview for DSM-IV-TR Axis I Disorders (SCID-I). As in a previous study [24] that investigated the link between BD and PD disorder, the diagnostic category for PD has been extended by lowering the thresholds required. In particular, three diagnostic categories were included: (1) PD with or without agoraphobia; (2) recurrent attacks, defined as three or more panic attacks (PAs) not meeting criteria for PD; and (3) limited symptoms attacks defined as three or more PAs with less than four of the thirteen panic symptoms required by the DSM-IV.

### 2.4. Temporal Relationship

The temporal relationship among BD, PD, and FM was investigated with the retrospective identification of the age-at-onset of the single threshold, full-blown illness. In particular, in BD-I patients the age-at-onset of the first manic or major depressive episodes was identified. In MDD and BD-II was defined as the onset of the first major depressive episode (because the onset of the first hypomania is difficult to trace accurately). The definition of age-at-onset of the major depressive episodes has been shown to be reliable [35], even considering the limitation of the retrospective assessment, such as recall bias. The age at onset of PD was the age at which the DSM-IV-TR criteria for PD were first met. The age at onset of FM was the age at which the patients first experience for at least three months a widespread pain, above and below the waist and on both sides of the body. In addition, axial skeletal pain (cervical spine or anterior chest or thoracic spine or low back) must be present.

Major events around which memories could be structured were identified to help the patients to recall the timing of the onset of a major depressive episode (MDE), PD, and FM. To maximize the accuracy of recall, we used the event history calendar, a conversational interviewing approach based on a tool designed to collect retrospective reports of events and the timing of their occurrences by asking the patients to reconstruct their geographical, work, and school positions throughout the investigated years.

Moreover, we assessed the time from symptom onset to diagnosis by asking the question “How long after you first mentioned your symptoms to a physician were you diagnosed with fibromyalgia?

### 2.5. Measures of Clinical Severity of Fibromyalgia

#### 2.5.1. Fibromyalgia Impact Questionnaire (FIQ)

The Italian version of FIQ [40] is a 10-item scale measuring FM-related symptoms. The first item contains 10 sub-items regarding physical functioning, each of which is rated on a 4-point Likert-type scale. Items 2 and 3 ask the patients to mark the number of days they felt well and the number of days they were unable to work because of FM symptoms. In items 4–10 the patients have to rate work difficulty, pain, fatigue, morning tiredness, stiffness, anxiety, and depression on horizontal linear scales marked in 10 increments. The maximum possible total score is 100. Higher scores indicate a greater impact of fibromyalgia on the functioning.

#### 2.5.2. The Fibromyalgia Assessment Status (FAS)

FAS [41] is an index that combines in a single measure (range: 0–10) the patient’s assessment of fatigue, sleep disturbances, and pain evaluated on the basis of the 16 non-articular sites listed on the Self-Assessment Pain Scale (SAPS).

#### 2.5.3. Health Assessment Questionnaire (HAQ)

HAQ [42] is a 20-item questionnaire investigating difficulties in performing eight daily-life activities categories (dressing and grooming, arising, eating, walking, hygiene, reach, grip, and outside activities). For each item, the patient is asked to rate the level of difficulty experienced over the preceding week in performing each activity using a 4-point scale ranging from 0 (no difficulty) to 3 (unable to perform). The final total HAQ score is the average score of the eight categories and ranges from 0 (no problem) to 3 (the worst score).

### 2.6. Statistical Analysis

A descriptive analysis was initially conducted, estimating the numbers and frequencies of the single threshold and subthreshold mood and panic disorders in patients with FM. Differences in demographic and clinical variables between the single disorders were explored using chi-square analyses for categorical variables and one-way ANOVAs (F tests) for numeric variables, both for mood disorders and panic disorders 

Subsequently, the sample was split according to the presence or absence of threshold and subthreshold comBD/PD, resulting in four groups of patients (two with and without threshold comBD/PD and two with and without threshold+subthreshold comBD/PD), which were then compared. Statistical methods consisted of Fisher’s exact test for the comparison of categorical data and Student’s *t*-test for dimensional variables. All statistics are two-tailed, and significance was set at a *p* value less than 0.05. Data were analyzed using R software (R Foundation for Statistical Computing, Vienna, Austria).

## 3. Results

### 3.1. Characteristics of the Sample

There were 118 patients with FM included in the analysis. The participants were of Caucasian ethnicity, predominantly females (90.7%), and their mean age was 45.7 ± 12 years. The mean age at onset of FM was 32.7 ± 12 years, while the mean duration of illness was 129.2 ± 114 months.

### 3.2. Mood Spectrum Diagnosis

The overall diagnostic, demographic, and clinical data are summarized in Table 1. 70 (59.3%) of FM patients were diagnosed as Bipolar II and only 17 (14.4%) as MinBD. Patients with no affective disorders were 31 (26.3%). The percentage of females was about 90% in all the three diagnostic groups, with no significant statistical difference. Further, demographic and clinical parameters did not statistically differ among the groups.

A comparison between Zurich and DSM-IV-TR classifications is shown in Table 2. All further analyses are based on the Zurich criteria.

### 3.3. Panic Spectrum Diagnosis

Of the 118 patients with FM entered this study, 83 (70.3%) were diagnosed with PD, 11 (9.3%) with recurrent PAs not meeting criteria for the disorder and a further 8 (6.8%) with limited symptoms PAs. FM patients without PD and subthreshold panic as defined above were 16 (13.6%). The four groups differed neither for socio-demographic parameters nor for age at onset, length of FM, and time to diagnosis (Table 3).

### 3.4. The Extent of BD/PD Comorbidity

**Figure 1:**
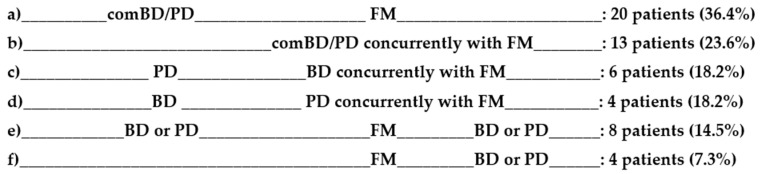
(**a**) Prevalence of threshold comBD/PD; (**b**) Prevalence of threshold + subthreshold comBD/PD.

The co-morbidity of mood disorders, substantially BD-II, and PD was seen in 46.6% (55/118) of FM patients (Figure 1a). When patients with minor bipolar disorder (MinBD) and subthreshold panic were included, the rate of co-morbidity raised to 68.6% (81/118) (Figure 1b).

### 3.5. The Temporal Sequencing of Threshold BD, PD and FM

**Figure 2:**
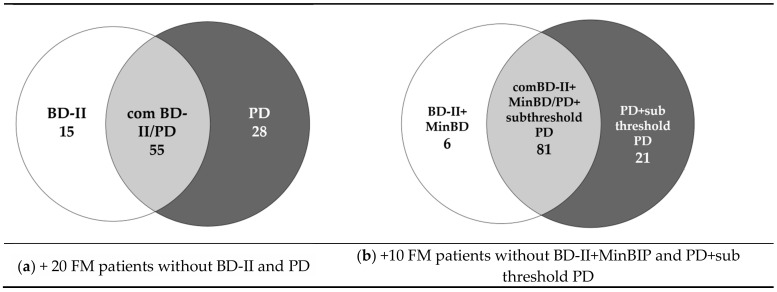
The temporal sequencing of threshold BD, PD, and FM.

In 43 patients out of 55 (78.2%) comBD/PD preceded or developed concurrently with FM. The temporal sequencing of threshold BD and PD is shown in Figure 2.

### 3.6. Comparison of Clinical Characteristics between the Threshold comBD/PD and no-comBD/PD

Table 4 shows a comparison of the demographic and clinical characteristics between the two groups. There were no statistically significant differences between the two groups in terms of age, sex, education level, civil status, duration, age at onset and time to diagnosis of FM, severity of FM symptoms and functional impairment.

The comparison of the threshold and subthreshold comBD/PD and no-comBD/PD yielded similar results (Supplementary Table S1).

## 4. Discussion

The study showed that in patients with FM there is a considerable rate (46.6%) of co-morbid BD and PD, which reaches the percentage of 68.6% when subthreshold presentations were included. This rate of co-morbid threshold BD and PD more than double that found in the general population by the Epidemiological Catchment Area study, which reported a lifetime prevalence for PD in BD of 20.8%, in turn, more than twice the rate of 10% observed in patients with MDD [20]. The US National Comorbidity Survey Replication [43], a nationally representative survey, distinguished individuals with a lifetime history of PAs without PD or agoraphobia (AG) (PA only), PAs with AG but not PD; (PA–AG), PD without AG (PD only), and PD with AG (PD–AG). These four diagnostic subgroups have comorbid BD I and II disorders in 7.1%, 15.5%, 14.4%, and 33% of cases, respectively, figures that are lower than those found in our study. A meta-analysis of 46 articles concluded that a pooled lifetime prevalence of PD is detectable in 16.8% of BD patients under treatment at psychiatric services [19].

Our finding indicated a relationship between a distinct subgroup of BD with comorbid PD and FM and are in agreement with a vast literature showing an association among higher rates of BD-II and PD and migraine, a recurrent headache disorder frequently observed in patients with FM [44], suggesting an association of comBD/PD and pain-related illnesses.

In a speculative vein, we hypothesize that the same genetic component underlying the co-occurrence of bipolar II disorders and PD could be a predisposing factor to the development of FM. This hypothesis is supported by the finding that comBD/PD appeared to precede or develop concurrently with FM in nearly 80% of our sample, and therefore it cannot be interpreted as reactive to the presence of symptoms and functional limitations produced by FM.

A complex interplay of biological and environmental factors could contribute to the co-occurrence of BD and PD in patients with FM. In particular, an imbalance in monoamine neurotransmission, mainly serotonin and norepinephrine, has been considered a pivotal mechanism in the pathophysiology of BP, PD [45,46], and FM [47,48]. Substance P (SP), a neuropeptide co-localized with monoamines which has long been known to be involved in regulating pain, has been recently found implicated in the modulation of stress, depression, and PD [49,50]. Finally, a number of studies suggested that inflammation, mainly due to an imbalance of pro- and anti-inflammatory cytokines, may be involved in the pathophysiology of BD [51], PD [52], and FM [53]. Environmental risk factors, such as early childhood adversity, were reported by patients with BD [54,55] PD [56], and FM [57] at rates higher than in general population.

As stated above, when subthreshold presentations of BD and PD have been considered, about 70% of FM patients in our sample presented this double comorbidity. A growing body of evidence highlights both the epidemiologic and clinical relevance of the milder form of BD and PD. The application of the subthreshold bipolarity diagnostic criteria in a growing number of population surveys has shown that as many as 11.2% of adults presented bipolar spectrum disorders [58]. In addition to the epidemiological consistency of subthreshold BD, it is well known that this population is prone to experiencing severe role impairment and negative outcomes (including co-morbidity with Ads, high risks of physical illnesses, and suicidal attempts), which are virtually equivalent to those occurring in BD-I and BD-II [59,60]. In FM patients, a growing body of studies has pointed out the centrality of subthreshold mood features, in particular those related to hypomanic symptoms [5,8,61] as well as behavioral phenotypes characterized by overactivity [62] and intense creative energy [63]. In agreement with these studies, in our sample high levels of hypomanic symptoms, energy and activity, as detected with HCL-32 checklist scores, were present across the threshold and subthreshold diagnostic categories of mood disorders and even in subjects without clinically identifiable mood disorders.

A population-based study showed that subthreshold panic is even more common (2.7%) than PD (1.7%), especially in women, and is associated with a high risk of developing psychiatric disorders functional impairment and work-related disability [64]. In the context of co-occurring psychiatric disorders, subthreshold PD has been associated with increased symptom severity, higher rates of comorbidity and suicidality, and poorer treatment response [32].

In our sample, FM patients with and without comBD/PD did not differ in demographic characteristics, as well as in clinical and severity parameters of FM. The present study did not evaluate the effects of depression and anxiety on single key symptoms of FM. In our population, all the patients recruited reported the presence of pain, fatigue, unrefreshing sleep, and cognitive impairment (rated as complaints of memory loss or impaired concentration but not with an objective neuropsychological test battery). For this reason, we rated the impact of FM symptoms with composite indices which quantify the various dimensions of the disease (FIQ, FAS, and HAQ,).

Overall, the effect of depression and anxiety on key FM symptoms is not fully elucidated. Several studies, but not all [2] demonstrated an association between high levels of depression and anxiety [65,66,67,68], as well as of current and past depressive and anxiety disorders [69] with more physical symptoms and poorer functioning in patients with FM. Among these studies, two [67,68] did not find a significant interaction effect between anxiety and depression on pain severity (i.e., anxiety and depression did not amplify the effect of the other).

Along with these results, we have to consider several cluster analysis investigations, [70,71,72] which sought to identify subsets of patients with fibromyalgia with similar symptom profiles, that identified clusters of patients with moderate FM symptoms and functional disabilities, as rated with FIQ, in the presence of high anxiety and depressive symptoms rates.

The results of cluster analysis studies and the evidence of the lack of an interaction effect of depression and anxiety on FM symptoms are consistent with our finding of similar clinical and functional impairment in patients with a different burden of depressive and anxiety symptoms and disorders.

It is possible to hypothesize that in FM patients the presence of comBD/PD does not relate to clinical or severity features (except the FM comorbidity) but could be a core characteristic of a part of FM patients attending to a tertiary care center. Some neuroimaging and physiological findings supported the view that mood/panic comorbidity may not be just a more severe version of the “pure” disorders, but it may reflect a complex interplay, with reciprocal influences, between mood and panic, resulting in a characteristic phenomenological and pathophysiological profile. In particular, depression/panic comorbidity displayed peculiar cerebral activation [73] and connectivity [74] patterns as well as different behavioral hypersensitivity to hypercapnia [75] in comparison with the single depressive and panic disorders.

Our finding is meaningful not only for the possible etiological perspective but also for its clinical implications. A large body of investigations has highlighted an elevated suicide risk in subjects with both threshold and subthreshold comBD/PD [76]. As several studies [77,78] supported the notion that individuals with FM have a significant risk of suicide, about 10 times that of the general population in females, [79] it can be assumed that a substantial part of this risk could be related to the presence of co-morbid threshold and subthreshold BD and PD. If this hypothesis is confirmed by further studies, early and targeted treatment of the psychiatric co-morbidity may have a preventive action against suicidal behavior in FM patients. 

Finally, our results raise questions about the proper pharmacological management of comorbid BD/PD in patients with FM. While in general antidepressants are the treatment of choice for PD and are widely used in FM for the control of pain, this might not be the case in patients with BD (at least not in monotherapy) because of the risk of medication-induced mood switch [80]. Unfortunately, no pharmacological randomized, controlled trials have been conducted in comorbid BD and PD disorders, even in patients without additional pathological conditions. However, despite the lack of evidence, there is a consensus that the primary aim of treatment is mood stabilization, and only following this prescription, treatment with other specific medications might be considered.

Our results should be considered in the context of several limitations. First, the study used a cross-sectional design, which precludes finding causal associations among the disorders. Therefore, longitudinal designs should be employed before causal inferences can be made. However, some inference regarding the directional and developmental pathways that connect the disorders can be made on the basis of temporal relationship evaluation showing that the vast majority (78%) of comBD/PD precede or develop concurrently with FM.

Second, the study did not include a control group, which avoids the comparison of the study group with a normal population and/or BD patients without FM. Third, the retrospective method of collecting data could impact the recall of symptoms that occurred many years ago. Fourth, given the relatively small sample size, it will be important to investigate this research question with larger samples. Finally, our sample is from a tertiary care clinic and the results may not be generalizable to all populations of FM individuals.

## Figures and Tables

**Table 1:** Mood disorders diagnosis, demographic, and clinical characteristics.

	Bipolar Spectrum Disorders			Unipolar Spectrum Disorders		No Mood Disorders	χ^2^ or F	*p*-Value
	Major Bipolar Spectrum		Minor Bipolar Spectrum	Major Unipolar Spectrum	Minor Unipolar Spectrum			
	BD-I	BD-II	MinBD	MDD	Dysthymia MinD or RBD			
Patients *n* (%)	0 (0)	70 (59.3)	17 (14.4)	0(0)	0(0)	31 (26.3)		
Sex-females *n* (%)		63 (90)	15 (88.2)			29 (93.5)	0.460	0.794
Age (years)		46.5 ± 11.5	45.8 ± 12.7			43.6 ± 12.9	0.614	0.543
Education (years)		11.7 ± 2.9	11.4 ± 4.3			11.4 ± 4	0.121	0.886
Marital Status Single Married Divorced/Widowed		13 45 12	4 11 2			7 17 7	1.358	0.850
Occupation Manager White-collar Blue-collar Unemployed		6 34 20 11	3 5 8 1			3 13 7 8	7.647	0.265
Age at Onset FM (years)		35.6 ± 12.2	33 ± 16.5			34 ± 14	0.322	0.726
Duration FM (months)		128.1 ± 105	162 ± 157.6			110.2 ± 109	1.068	0.347
Time to FM diagnosis		102.2 ± 98.5	96.1 ± 122.6			72.4 ± 100	0.788	0.458
HCL-32 total sc.(months)		18.9 ± 4.1	16.9 ± 4.9			16.8 ± 4.6	2.744	0.69
HCL-32 ≥ 14 Yes No		57 5	13 3			20 6	3.998	0.135
BMI		24 ± 3.7	24.9 ± 3.9			23.9 ± 4	0.347	0.308

Values are mean (SD) or number (*n*) and percentage (%). Chi-square test was used for categorical variables and ANOVA was used for continuous variables. FM: Fibromyalgia; BD-I: Bipolar I disorder; BD-II: Bipolar II disorder; MDD: Major depression disorder; MinBD: Minor Bipolar disorder; MinD: Minor Depression; RDB: Recurrent Brief Depression; HCL-32: Hypomania Symptom Checklist; BMI: Body Mass Index.

**Table 2:** Diagnoses according to Diagnostic and Statistical Manual of Mental Disorders-IV-Text Revision (DSM-IV-TR) and Zurich criteria.

	Major Bipolar Spectrum		Minor Bipolar Spectrum	
	MDE with Hypomanic Features/BD-II		Minor Bipolar Disorder (MinBD):	
	*n* = 70		*n* = 17	
DSM IV-TR	BD-II *n* = 34	BD-NOS *n* = 36	BD-NOS *n* = 10	Cyclothymic Disorder *n* = 7
ZurichCriteria	MDE+ Zurich Criteria Hypomanic Syndrome (BD-II) *n* = 50	MDE+ Hypomanic Symptoms (BD-II) *n* = 20	Minor Bipolar Disorder (MinBD) *n* = 17	

MDE: Major depressive episode; BD-NOS: Bipolar disorder not otherwise specified; BIP-II: Bipolar II disorder.

**Table 3:** Panic disorder (PD) and subthreshold panic, demographic, and clinical characteristics.

	Threshold PD	Sub-Threshold PD		No Threshold or Subthreshold PD	χ^2^ or F	*p* Value
	PD	Recurrent PAs	Limited Symptoms PAs			
Patients *n* (%)	83 (70.3)	11 (9.3)	8 (6.8)	16 (13.6)		
Sex-females *n* (%)	77 (92.7)	10 (91)	7 (87.5)	13 (81.2)	2.209	0.530
Age (years)	45.7 ± 13	42.9 ± 10.1	43.2 ± 4.6	48.7 ± 10.6	0.629	0.598
Education (years)	11.4 ± 3.2	12.2 ± 3.8	12.4 ± 3	11.6 ± 4.2	0.318	0.812
Marital status Single Married Divorced/Widowed	15 55 13	4 3 4	3 4 1	2 11 3	8.410	0.210
Occupation Manager White-collar Blue-collar Unemployed	10 33 26 14	2 3 4 2	0 6 2 0	0 10 3 3	9.81	0.366
Age at Onset of FM (years)	34.2 ± 14.1	31.6 ± 8.5	34.2 ± 7.6	40.1 ± 12.5	1.112	0.348
Duration of FM (months)	136.9 ± 121.2	132.3 ± 112.6	107.2 ± 104.4	97.2 ± 78.8	0.608	0.611
Time to FM diagnosis	102.1 ± 105.1	111 ± 114	73.9 ± 102.3	50.4 ± 69.4	1.212	0.309
BMI	24.4 ± 3.9	22.6 ± 3.8	23.8 ± 4.1	24.2 ± 3.4	0.564	0.640

Values are mean (SD) or number (*n*) and percentage (%). Chi-square test was used for categorical variables and ANOVA was used for continuous variables. BMI: Body Mass Index.

**Table 4:** Comparison between FM patients with and without comorbid threshold BD/PD.

	COMORBID BD/PD	NoCOMORBID BD/PD	t or χ^2^	*p*-Value
Number of patients *n* (%)	55 (46.6%)	63 (53.4%)		
Sex *n* (%) Male Female	5 (9.1) 50 (90.9)	6 (9.5) 57 (90.5)	0.007	1.00
Age (years)	47.4 ± 11.9	44.2 ± 12.1	1.42	0.156
Education (years)	11.7 ± 3.1	11.5 ± 3.7	0.257	0.798
Marital status *n* (%) Single Married Divorced/widowed	9 (16.4) 37 (67.2) 9 (16.4)	15 (23.9) 36 (57.1) 12(19)	1.406	0.495
Occupation *n* (%) Manager White-collar Blue-collar Unemployed	8 (14.5) 17 (31) 24 (43.5) 6 (11)	11(17.5) 18 (28.6) 28 (44.4) 6 (9.5)	0.269	0.966
Age at Onset of FM (years)	36.7 ± 12.4	33.1 ± 13.7	1.440	0.153
Duration of FM (months)	124.9 ± 101.3	133 ± 125.4	−0.377	0.707
Time to FM diagnosis (months)	100.82 ± 93.5	87.98 ± 110.1	0.643	0.522
BMI	24.2 ± 3.6	24 ± 4	0.231	0.818
VAS pain	8.1 ± 0.9	8.2 ± 1.3	−0.210	0.834
FIQ	78 ± 10.3	76 ± 12.7	0.849	0.398
FAS	7.6 ± 1.2	7.6 ± 1.05	−0.272	0.786
HAQ	1.1 ± 0.4	1.02 ± 0.4	0.165	0.311

Values are mean (SD) or number (*n*) and percentage (%). Chi-square test was used for categorical variables and t-test was used for continuous variables. BMI: Body Mass Index; VAS pain: Visual Analog Scale for Pain (included in FIQ); FIQ: Fibromyalgia Impact Questionnaire; FAS Fibromyalgia Assessment Scale; HAQ: Health Assessment Questionnaire.

## Supplementary Materials

The following are available online at https://www.mdpi.com/2077-0383/9/11/3619/s1, Table S1. Comparison between FM patients with and without comorbid threshold and subthreshold BD/PD

## References

[1] Raphael K.G., Janal M.N., Nayak S., Schwartz J.E., Gallagher R.M. (2006). Psychiatric comorbidities in a community sample of women with fibromyalgia. Pain.

[2] Thieme K., Turk D.C., Flor H. (2004). Comorbid depression and anxiety in fibromyalgia syndrome: Relationship to somatic and psychosocial variables. Psychosom. Med..

[3] Gracely R.H., Ceko M., Bushnell M.C. (2012). Fibromyalgia and depression. Pain Res. Treat..

[4] Aguglia A., Salvi V., Maina G., Rossetto I., Aguglia E. (2011). Fibromyalgia syndrome and depressive symptoms: Comorbidity and clinical correlates. J. Affect. Disord..

[5] Alciati A., Sarzi-Puttini P., Batticciotto A., Torta R., Gesuele F., Atzeni F., Angst J. (2012). Overactive lifestyle in patients with fibromyalgia as a core feature of bipolar spectrum disorder. Clin. Exp. Rheumatol..

[6] Kudlow P.A., Rosenblat J.D., Weissman C.R., Cha D.S., Kakar R., McIntyre R.S., Sharma V. (2015). Prevalence of fibromyalgia and co-morbid bipolar disorder: A systematic review and meta-analysis. J. Affect. Disord..

[7] Arnold L.M., Hudson J.I., Hess E.V., Ware A.E., Fritz D.A., Auchenbach M.B., Starck L.O., Keck P.E. (2004). Family study of fibromyalgia. Arthritis Rheumatol..

[8] Carta M.G., Cardia C., Mannu F., Intilla G., Hardoy M.C., Anedda C., Ruggero V., Fornasier D., Cacace E. (2006). The high frequency of manic symptoms in fibromyalgia does influence the choice of treatment?. Clin. Pract. Epidemiol. Ment. Health.

[9] Arnold L.M., Hudson J.I., Keck P.E., Auchenbach M.B., Javaras K.N., Hess E.V. (2006). Comorbidity of fibomyalgia and psychiatric disorders. J. Clin. Psychiatry.

[10] Arnold L.M., Leon T., Whalen E., Barrett J. (2010). Relationships among pain and depressive and anxiety symptoms in clinical trials of pregabalin in fibromyalgia. Psychosomatics.

[11] Uguz F., Çiçek E., Salli A., Karahan A.Y., Albayrak I., Kaya N., Uğurlu H. (2010). Axis I and Axis II psychiatric disorders in patients with fibromyalgia. Gen. Hosp. Psychiatry.

[12] Kessler R.C., Petukhova M., Sampson N.A., Zaslavsky A.M., Wittchen H.U. (2012). Twelve-month and lifetime prevalence and lifetime morbid risk of anxiety and mood disorders in the United States. Int. J. Methods Psychiatr. Res..

[13] Gormsen L., Rosenberg R., Bach F.W., Jensen T.S. (2010). Depression, anxiety, health-related quality of life and pain in patients with chronic fibromyalgia and neuropathic pain. Eur. J. Pain.

[14] Tander B., Cengiz K., Alayli G., Ilhanli I., Canbaz S., Canturk F. (2008). A comparative evaluation of health related quality of life and depression in patients with fibromyalgia syndrome and rheumatoid arthritis. Rheumatol. Int..

[15] Stasi C., Nisita C., Cortopassi S., Corretti G., Gambaccini D., De Bortoli N., Fani B., Simonetti N., Ricchiuti A., Dell’Osso L. (2017). Subthreshold psychiatric psychopathology in functional gastrointestinal disorders: Can it be the bridge between gastroenterology and psychiatry?. Gastroenterol. Res. Pract..

[16] Provencher M.D., Guimond A.J., Hawke L.D. (2012). Comorbid anxiety in bipolar spectrum disorders: A neglected research and treatment issue?. J. Affect. Disord..

[17] Frank E., Cyranowski J.M., Rucci P., Shear M.K., Grochocinski V.J., Kostelnik B., Kupfer D.J. (2016). Clinical significance of lifetime panic spectrum symptoms in the treatment of patients with bipolar I disorder. Arch. Gen. Psychiatry.

[18] Henry C., Van Den Bulke D., Bellivier F., Etain B., Rouillon F., Leboyer M. (2003). Anxiety disorders in 318 bipolar patients: Prevalence and impact on illness severity and response to mood stabilizer. J. Clin. Psychiatry.

[19] Nabavi B., Mitchell A.J., Nutt D.A. (2015). Lifetime prevalence of comorbidity between bipolar affective disorder and anxiety disorders: A meta-analysis of 52 interview-based studies of psychiatric population. EBioMedicine.

[20] Chen Y.-W., Dilsaver S.C. (1995). Comorbidity of panic disorder in bipolar illness: Evidence from the epidemiologic catchment area survey. Am. J. psychiatry.

[21] Pini S., Cassano G.B., Simonini E., Savino M., Russo A., Montgomery S.A. (1997). Prevalence of anxiety disorders comorbidity in bipolar depression, unipolar depression and dysthymia. J. Affect. Disord..

[22] Boylan K.R., Bieling P.J., Marriott M., Begin H., Young L.T., MacQueen G.M. (2004). Impact of comorbid anxiety disorders on outcome in a cohort of patients with bipolar disorder. J. Clin. Psychiatry.

[23] Savino M., Perugi G., Simonini E., Soriani A., Cassano G.B., Akiskal H.S. (1993). Affective comorbidity in panic disorder: Is there a bipolar connection?. J. Affect. Disord..

[24] Doughty C.J., Wells J.E., Joyce P.R., Olds R.J., Walsh A.E.S. (2004). Bipolar-panic disorder comorbidity within bipolar disorder families: A study of siblings. Bipolar Disord..

[25] Angst J., Gamma A., Bowden C.L., Azorin J.M., Perugi G., Vieta E., Young A.H. (2013). Evidence-based definitions of bipolar-I and bipolar-II disorders among 5,635 patients with major depressive episodes in the Bridge Study: Validity and comorbidity. Eur. Arch. Psychiatry Clin. Neurosci..

[26] MacKinnon D.F., McMahon F.J., Simpson S.G., McInnis M.G., DePaulo J.R. (1997). Panic disorder with familial bipolar disorder. Biol. Psychiatry.

[27] MacKinnon D.F., Xu J., McMahon F.J., Simpson S.G., Stine O.C., Mclnnis M.G., DePaulo J.R. (1998). Bipolar disorder and panic disorder in families: An analysis of chromosome 18 data. Am. J. Psychiatry.

[28] McMahon F.J., Simpson S.G., McInnis M.G., Badner J.A., MacKinnon D.F., DePaulo R. (2001). Linkage of bipolar disorder to chromosome 18q and the validity of bipolar II disorder. Arch. Gen. Psychiatry.

[29] Stine O.C., Xu J., Koskela R., McMahon F.J., Gschwend M., Friddle C., Clark C.D., McInnis M.G., Simpson S.G., Breschel T.S. (1995). Evidence for linkage of bipolar disorder to chromosome 18 with a parent-of-origin effect. Am. J. Hum. Genet..

[30] MacKinnon D.F., Zandi P.P., Cooper J., Potash J.B., Simpson S.G., Gershon E., Nurnberger J., Reich T., DePaulo J.R. (2002). Comorbid bipolar disorder and panic disorder in families with a high prevalence of bipolar disorder. Am. J. Psychiatry.

[31] Batelaan N.M., de Graaf R., Spijker J., Smit J.H., van Balkom A.J.L.M., Vollebergh W.A.M., Beekman A.T. (2010). The course of panic attacks in individuals with panic disorder and subthreshold panic disorder: A population-based study. J. Affect. Disord..

[32] Pané-Farré C.A., Fenske K., Stender J.P., Meyer C., John U., Rumpf H.J., Hapke U., Hamm A.O. (2013). Sub-threshold panic attacks and agoraphobic avoidance increase comorbidity of mental disorders: Results from an adult general population sample. J Anxiety Disord..

[33] Wolfe F., Smythe H.A., Yunus M.B., Bennett R.M., Bombardier C., Goldenberg D.L., Tugwell P., Campbell S.M., Abeles M., Clark P. (1990). The American college of rheumatology 1990 criteria for the classification of fibromyalgia. Report of the multicenter criteria committee. Arthritis Rheumatol..

[34] First M.B., Spitzer R.L., Gibbon M., Williams J. (2001). Structured Clinical Interview for DSM-IV-TR Axis I Disorders.

[35] Benazzi F., Akiskal H.S. (2003). Refining the evaluation of bipolar II: Beyond the strict SCID-CV guidelines for hypomania. J. Affect. Disord..

[36] Angst J., Gamma A., Benazzi F., Ajdacic V., Eich D., Rössler W. (2003). Toward a re-definition of subthreshold bipolarity: Epidemiology and proposed criteria for bipolar-II, minor bipolar disorders and hypomania. J. Affect. Disord..

[37] Judd L.L., Akiskal H.S. (2003). The prevalence and disability of bipolar spectrum disorders in the US population: Re-analysis of the ECA database taking into account subthreshold cases. J. Affect. Disord..

[38] Angst J., Adolfsson R., Benazzi F., Gamma A., Hantouche E., Meyer T.D., Skeppar P., Vieta E., Scott J. (2005). The HCL-32: Towards a self-assessment tool for hypomanic symptoms in outpatients. J. Affect. Disord..

[39] Meyer T.D., Schrader J., Ridley M., Lex C. (2014). The Hypomania Checklist (HCL)—Systematic review of its properties to screen for bipolar disorders. Compr. Psychiatry.

[40] Burckhardt C.S., Clark S.R., Bennett R.M. (1991). The fibromyalgia impact questionnaire: Development and validation. J. Rheumatol..

[41] Salaffi F., Sarzi-Puttini P., Girolimetti R., Gasparini S., Atzeni F., Grassi W. (2009). Development and validation of the self-administered fibromyalgia assessment status: A disease-specific composite measure for evaluating treatment effect. Arthritis Res. Ther..

[42] Bruce B., Fries J.F. (2003). The Stanford health assessment questionnaire: Dimensions and practical applications. Health Qual. Life Outcomes.

[43] Kessler R.C., Wai T.C., Jin R., Ruscio A.M., Shear K., Walters E.E. (2006). The epidemiology of panic attacks, panic disorder, and agoraphobia in the National Comorbidity Survey Replication. Arch. Gen. Psychiatry.

[44] Fasmer O.B., Oedegaard K.J. (2001). Clinical characteristics of patients with major affective disorders and comorbid migraine. World J. Biol. Psychiatry.

[45] Rotondo A., Mazzanti C., Dell’Osso L., Rucci P., Sullivan P., Bouanani S., Gonnelli C., Goldman D., Cassano G.B. (2002). Catechol o-methyltransferase, serotonin transporter, and tryptophan hydroxylase gene polymorphisms in bipolar disorder patients with and without comorbid panic disorder. Am. J. Psychiatry.

[46] Campos S.B., Miranda D.M., Souza B.R., Pereira P.A., Neves F.S., Tramontina J., Kapczinski F., Romano-Silva M.A., Correa H. (2011). Association study of tryptophan hydroxylase 2 gene polymorphisms in bipolar disorder patients with panic disorder comorbidity. Psychiatr. Genet..

[47] Benson C., Mifflin K., Kerr B., Jesudasan S.J.B., Dursun S., Baker G. (2015). Biogenic amines and the amino acids GABA and glutamate: Relationships with pain and depression. Pain in Psychiatric Disorders.

[48] Harvey M., Gagné B., Labbé M., Barden N. (2007). Polymorphisms in the neuronal isoform of tryptophan hydroxylase 2 are associated with bipolar disorder in French Canadian pedigrees. Psychiatr. Genet..

[49] Herpfer I., Lieb K.P. (2005). Substance P receptor antagonists in psychiatry: Rationale for development and therapeutic potential. CNS Drugs.

[50] Perna G., Schruers K., Alciati A., Caldirola D. (2015). Novel investigational therapeutics for panic disorder. Expert Opin. Investig. Drugs..

[51] Muneer A. (2016). The neurobiology of bipolar disorder: An integrated approach. Chonnam Med. J..

[52] Perna G., Iannone G., Alciati A., Caldirola D. (2016). Are anxiety disorders associated with accelerated aging? A focus on neuroprogression. Neural Plast..

[53] Maes M. (2009). Inflammatory and oxidative and nitrosative stress pathways underpinning chronic fatigue, somatization and psychosomatic symptoms. Curr. Opin. Psychiatry.

[54] Bergink V., Larsen J.T., Hillegers M.H.J., Dahl S.K., Stevens H., Mortensen P.B., Petersen L., Munk-Olsen T. (2016). Childhood adverse life events and parental psychopathology as risk factors for bipolar disorder. Transl. Psychiatry.

[55] Dell’Osso M.C., Conversano C., Lensi E., Granchi F., Consoli G., Faravelli L., Rotella F., Sarno N., Faravelli C. (2008). Agorafobia: Un problema irrisolto. Ital. J. Psychopathol..

[56] Perna G., Alciati A., Prestia D., Torti T., Nemeroff C.B. (2013). Is there a link between child abuse and neglect and anxiety disorders?. Minerva Psichiatr..

[57] Alciati A., Atzeni F., Grassi M., Caldirola D., Riva A., Sarzi-Puttini P., Perna G. (2017). Childhood adversities in patients with fibromyalgia: Are they related to comorbid lifetime major depression?. Clin. Exp. Rheumatol..

[58] Angst J., Merikangas J.A.K.R., Meter L.C.A., Van Rössler V.A.G.W. (2018). Bipolar spectrum in major depressive disorders. Eur. Arch. Psychiatry Clin. Neurosci..

[59] Merikangas K.R., Jin R., He J., Kessler R.C., Lee S., Sampson N.A., Viana M.C., Andrade L.H., Hu C., Karam E.G. (2011). Prevalence and correlates of bipolar spectrum disorder in the world mental health survey initiative. Arch. Gen. Psychiatry.

[60] Nusslock R., Frank E. (2011). Subthreshold bipolarity: Diagnostic issues and challenges. Bipolar Disord..

[61] Piccinni A., Bazzichi L., Marazziti D., Veltri A., Bombardieri S., Conversano C., Ciapparelli A., Dell’Osso L. (2011). Subthreshold mood symptoms in patients with fibromyalgia and rheumatoid arthritis. Clin. Exp. Rheumatol..

[62] Van Houdenhove B., Neerinckx E., Onghena P., Lysens R., Vertommen H. (2001). Premorbid “overactive” lifestyle in chronic fatigue syndrome and fibromyalgia: An etiological factor or proof of good citizenship?. J. Psychosom. Res..

[63] Schmechel D.E., Edwards C.L. (2012). Fibromyalgia, mood disorders, and intense creative energy: A1AT polymorphisms are not always silent. Neurotoxicology.

[64] Skapinakis P., Lewis G., Davies S., Brugha T., Prince M., Singleton N. (2011). Panic disorder and subthreshold panic in the UK general population: Epidemiology, comorbidity and functional limitation. Eur. Psychiatry.

[65] Kurtze N., Gundersen K.T., Svebak S. (1998). The role of anxiety and depression in fatigue and patterns of pain among subgroups of fibromyalgia patients. Br. J. Med. Psychol..

[66] White K.P., Nielson W.R., Harth M., Ostbye T., Speechley M. (2002). Chronic widespread musculoskeletal pain with or without fibromyalgia: Psychological distress in a representative community adult sample. J. Rheumatol..

[67] Alok R., Das S.K., Agarwal G.G., Salwahan L., Srivastava R. (2011). Relationship of severity of depression, anxiety and stress with severity of fibromyalgia. Clin. Exp. Rheumatol..

[68] Hadlandsmyth K., Dailey D.L., Rakel B.A., Zimmerman M.B., Vance C.G., Merriwether E.N., Chimenti R.L., Geasland K.M., Crofford L.J., Sluka K.A. (2020). Somatic symptom presentations in women with fibromyalgia are differentially associated with elevated depression and anxiety. J. Health Psychol..

[69] Walker E.A., Keegan D., Gardner G., Sullivan M., Katon W.J., Bernstein D. (1997). Psychosocial factors in fibromyalgia compared with rheumatoid arthritis: I. Psychiatric diagnoses and functional disability. Psychosom. Med..

[70] Wilson H.D., Robinson J.P., Turk D.C. (2009). Toward the identification of symptom patterns in people with fibromyalgia. Arthritis Rheumatol..

[71] Vincent A., Hoskin T.L., Whipple M.O., Clauw D.J., Barton D.L., Benzo R.P., Williams D.A. (2014). OMERACT-based fibromyalgia symptom subgroups: An exploratory cluster analysis. Arthritis Res. Ther..

[72] Salaffi F., Mozzani F., Draghessi A., Atzeni F., Catellani R., Ciapetti A., Di Carlo M., Sarzi-Puttini P. (2016). Identifying the symptom and functional domains in patients with fibromyalgia: Results of a cross-sectional Internet-based survey in Italy. J. Pain Res..

[73] Gorka S.M., Huggins A.A., Fitzgerald D.A., Nelson B.D., Phan K.L., Shankman S.A. (2014). Neural response to reward anticipation in those with depression with and without panic disorder. J. Affect. Disord..

[74] Lueken U., Straube B., Yang Y., Hahn T., Beesdo-Baum K., Wittchen H.U., Konrad C., Ströhle A., Wittmann A., Gerlach A.L. (2015). Separating depressive comorbidity from panic disorder: A combined functional magnetic resonance imaging and machine learning approach. J. Affect. Disord..

[75] Verburg K., Klaassen T., Pols H., Griez E. (1998). Comorbid depressive disorder increases vulnerability to the 35% carbon dioxide (CO2) challenge in panic disorder patients. J. Affect. Disord..

[76] Kilbane E.J., Gokbayrak N.S., Galynker I., Cohen L., Tross S. (2009). A review of panic and suicide in bipolar disorder: Does comorbidity increase risk?. J. Affect. Disord..

[77] Lan C.C., Tseng C.H., Chen J.H., Lan J.L., Wang Y.C., Tsay G.J., Hsu C.Y. (2016). Increased risk of a suicide event in patients with primary fibromyalgia and in fibromyalgia patients with concomitant comorbidities. Medicine (Baltimore).

[78] Tang N.K.Y., Crane C. (2006). Suicidality in chronic pain: A review of the prevalence, risk factors and psychological links. Psychol. Med..

[79] Dreyer L., Kendall S., Danneskiold-Samsøe B., Bartels E.M., Bliddal H. (2010). Mortality in a cohort of Danish patients with fibromyalgia: Increased frequency of suicide. Arthritis Rheumatol..

[80] Preti A., Vrublevska J., Veroniki A.A., Huedo-Medina T.B., Kyriazis O., Fountoulakis K.N. (2018). Prevalence and treatment of panic disorder in bipolar disorder: Systematic review and meta-analysis. Evid. Based Ment. Health.

