# Supplementary material for: The Co-Morbidity between Bipolar and Panic Disorder in Fibromyalgia Syndrome
Source: J Clin Med. 2020 Nov 10;9(11):3619. doi: 10.3390/jcm9113619 (PMC7697979; doi:10.3390/jcm9113619)
Supplement: Supplementary file 1 [file jcm-09-03619-s001.pdf]

Table S1. Comparison between FM patients with and without comorbid threshold and subthreshold BD/PD

	CO-MORBID THRESHOLD AND SUBTHRESHOLD BD AND PD	NO CO-MORBID THRESHOLD SUBTHRESHOLD BD AND PD	t or X^2	p-value
Number of patients N.(%)	81 (68.6)	37 (31.4)		
Sex N.(%)				
- Male	7(8.6)	4 (10.8)	.141	.739
- Female	74 (91.4)	33 (89.2)		
Age (years)	46.7±11.7	43.4 ±12.5	1.35	.177
Education (years)	11.5±3.2	11.8±3.8	-.450	.654
Marital status N.(%)				
- Single	16(19.8)	8(21.6)		
- Married	52(64.2)	21(56.8)	.714	.700
- Divorced/widowed	13(16)	8(21.6)		
Occupation N.(%)				
- Manager	9(11.1)	3(8.1)		
- White collar	33(40.7)	19(51.4)	3.991	.262
- Blue collar	28(34.6)	7(18.9)		
- Unemployed	11(13.6)	8(21.6)		
Age at Onset of FM (years)	35.4 ± 13.1	36.6 ± 13.6	.687	.494
Duration of FM (months)	134.9 ± 117.6	116.7 ± 107.2	.791	.431
Time to FM diagnosis	103 ± 103.5	73.31 ± 97.86	-1.378	.171
BMI	24.1 ± 3.7	24.1 ± 4	.075	.940
VAS pain	8.1 ± 1.02	8.2 ± 0.9	-.474	.636
FIQ	77.3 ± 11.3	76.1 ± 12.6	.456	.649
FAS	7.5 ± 1.2	7.7 ± 1.1	.403	.688
HAQ	1.06 ± 0 .4	1.07 ± 0.5	-.065	.948

Values are mean (SD) or number (N) and percentage (%). Chi square test was used for categorical variables,and t-test was used for continuous variables.

BMI: Body Mass Index; VAS pain: Visual Analog Scale for Pain (included in FIQ); FIQ: Fibromyalgia Impact Questionnaire; FAS Fibromyalgia Assessment Scale; HAQ: Health Assessment Questionnaire
